# H3.1/3.2 regulate the initial progression of the gene expression program

**DOI:** 10.1093/nar/gkae214

**Published:** 2024-04-03

**Authors:** Satoshi Funaya, Yusuke Takahashi, Masataka G Suzuki, Yutaka Suzuki, Fugaku Aoki

**Affiliations:** Department of Computational Biology and Medical Sciences, The University of Tokyo, Kashiwa 277-8562, Japan; Department of Integrated Biosciences, Graduate School of Frontier Sciences, The University of Tokyo, Kashiwa 277-8562, Japan; Department of Integrated Biosciences, Graduate School of Frontier Sciences, The University of Tokyo, Kashiwa 277-8562, Japan; Department of Integrated Biosciences, Graduate School of Frontier Sciences, The University of Tokyo, Kashiwa 277-8562, Japan; Department of Computational Biology and Medical Sciences, The University of Tokyo, Kashiwa 277-8562, Japan; Department of Computational Biology and Medical Sciences, The University of Tokyo, Kashiwa 277-8562, Japan; Department of Integrated Biosciences, Graduate School of Frontier Sciences, The University of Tokyo, Kashiwa 277-8562, Japan

## Abstract

In mice, transcription from the zygotic genome is initiated at the mid-one-cell stage, and occurs promiscuously in many areas of the genome, including intergenic regions. Regulated transcription from selected genes is established during the two-cell stage. This dramatic change in the gene expression pattern marks the initiation of the gene expression program and is essential for early development. We investigated the involvement of the histone variants H3.1/3.2 in the regulation of changes in gene expression pattern during the two-cell stage. Immunocytochemistry analysis showed low nuclear deposition of H3.1/3.2 in the one-cell stage, followed by a rapid increase in the late two-cell stage. Where chromatin structure is normally closed between the one- and two-cell stages, it remained open until the late two-cell stage when H3.1/3.2 were knocked down by small interfering RNA. Hi-C analysis showed that the formation of the topologically associating domain was disrupted in H3.1/3.2 knockdown (KD) embryos. Promiscuous transcription was also maintained in the late two-cell stage in H3.1/3.2 KD embryos. These results demonstrate that H3.1/3.2 are involved in the initial process of the gene expression program after fertilization, through the formation of a closed chromatin structure to execute regulated gene expression during the two-cell stage.

## Graphical Abstract

**Graphical Abstract:**
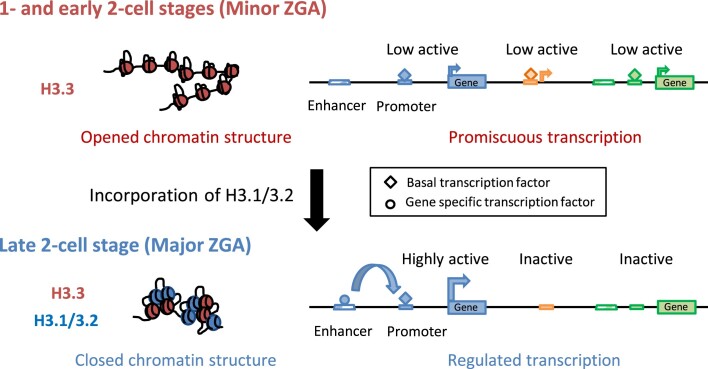

## Introduction

The development in organism proceeds according to a gene expression program. In mice, the zygotic genome is silent for a period after fertilization. Transcription is initiated during the mid-one-cell stage and transcriptional activity gradually increases until the late two-cell stage; this process termed zygotic gene activation (ZGA) (1,2). ZGA consists of two transcriptional activation phases. During the first wave of genome activation from the one-cell stage to the early two-cell stage, transcription occurs promiscuously over a large part of the genome, including intergenic regions, independent of enhancers (3–6); this process is termed minor ZGA (7). The second wave occurs during the mid- to late two-cell stage, in which the gene expression patterns change dramatically compared to the first wave through selective transcription depending on enhancers; this process is termed the major ZGA (3–6). Minor ZGA appears to be an indispensable prerequisite for major ZGA, because transient inhibition of transcription in the one-cell stage attenuates regulated transcription in the late two-cell stage (8). Thus, transcription is established in the zygotic genome through a change from promiscuous to regulated expression patterns during the two-cell stage. This change is the first process in the gene expression program after fertilization.

This change appears to be associated with a change in chromatin structure, which is dramatically closed during the transition from minor to major ZGA (7,9–11). Because one of the roles of enhancers is to open the chromatin structure to allow transcription factors to access gene promoters (12), an open structure appears to allow for enhancer-independent, promiscuous transcription during minor ZGA, whereas a closed structure creates a transcriptionally repressive state, in which enhancers are required for the process of selective transcription from specific genes during major ZGA (3–5,7,13). The second round of DNA replication in the two-cell stage has been suggested to be involved in changes in the chromatin structure and gene expression pattern during the transition from minor to major ZGA. When the second round of DNA replication is inhibited by treatment with aphidicolin, a DNA polymerase inhibitor, the chromatin structure remains open (10), enhancer-independent transcription occurs (3,14–16), and some marker genes of minor ZGA are still expressed in the late two-cell stage (17). Although these findings suggest that a chromatin structural change is involved in the progression of the gene expression program during the two-cell stage, the molecular mechanism regulating this change has not yet been elucidated.

Several variants of histone H3 play important roles in the regulation of chromatin structure. The variants H3.1, H3.2, and H3.3 are mainly reported in mammals. H3.1 and H3.2 are more likely to be modified by H3K9me2 and K27me2/3, which are involved in the formation of closed chromatin structure and are enriched in heterochromatin or euchromatin regions, where transcriptional levels are low (18–20). H3.1 and 3.2 are incorporated into chromatin in a process that is dependent on DNA replication (21). H3.3 is more likely to be modified by H3K4me3 and K27ac, which are involved in the formation of an open chromatin structure, and is enriched in euchromatin regions, where transcriptional levels are high, except in telomere regions (19,20,22). H3.3 is incorporated into chromatin in a DNA replication-independent manner (23). Previous studies have reported that the nuclear deposition of H3.1/3.2 occurs at extremely low levels in one-cell-stage embryos and increases dramatically during the two-cell stage, when the chromatin structure is closed (24,25). However, the roles of H3.1/3.2 in these changes in chromatin structure and gene expression between the two ZGA stages remain to be clarified.

In this study, we investigated the involvement of the histone variants H3.1/3.2 in the mechanisms regulating changes in chromatin structure and gene expression during minor and major ZGA by knocking down H3.1/3.2. Our results provide insights into the mechanisms underlying the early stages of the gene expression program.

## Materials and methods

### Collection and culture of oocytes and embryos

All experiments using mice were reviewed and approved by the University of Tokyo Institutional Animal Care and Use Committee and were performed in accordance with the Guiding Principles for the Care and Use of Laboratory Animals.

Fully grown oocytes were collected from 8- to 12-week-old B6D2F1 female mice (Japan SLC, Hamamatsu, Japan). Ovaries were collected from mice at 45–48 h after injection with 7.5 I.U. pregnant mare serum gonadotropin (PMSG; ASKA Pharmaceutical, Tokyo, Japan), and transferred to HEPES-buffered K+-modified simplex optimized medium (KSOM) (26) containing 0.2 mM 3-isobutyl-1-methylxanthine (IBMX; I5879, Sigma-Aldrich, St. Louis, MO, USA). Then the ovaries were punctured with a 30-gauge needle and only fully grown oocytes were collected.

Metaphase II (MII) stage oocytes were obtained from the ampulla of the oviduct in 3-week-old B6D2F1 female mice injected with 6 I.U. PMSG and at 48 h later with 7.5 I.U. human chorionic gonadotropin (ASKA Pharmaceutical). Sperm were collected from the cauda epididymis of adult Institute of Cancer Research (ICR) male mice (Japan SLC) and cultured in human tubal fluid medium (27) for *in vitro* fertilization. To obtain embryos, MII-stage oocytes were inseminated with spermatozoa pre-cultured for 2 h. After 6–9 h, the embryos were transferred to KSOM medium, washed with KSOM medium to remove surrounding sperm and cumulus cells, and cultured at 38°C in an atmosphere of 5% CO_2_ and 95% air.

### Semi-quantitative reverse-transcription PCR (RT-PCR) and qPCR

RNA extraction from MII-stage oocytes and embryos using ISOGEN (311-02501, Nippon Gene, Tokyo, Japan), reverse-transcription with random hexamer followed by semi-quantitative PCR, and qPCR were performed as described previously (5,28). The PCR primers and conditions are listed in Supplementary Table S1.

### Microinjection

Microinjection was performed using an inverted microscope (Eclipse TE300, Nikon, Tokyo, Japan), a micromanipulator (MO-202U, Narishige, Tokyo, Japan), and a microinjector (IM300, Narishige). Injection into fully grown oocytes was performed to obtain H3.1/3.2 KD embryos. Fully grown oocytes in HEPES-buffered KSOM medium containing 0.2 mM IBMX were injected with 10 pl Stealth RNAi siRNA against common sequences of H3.1 and 3.2 (H3.1/3.2; 10 μM; Thermo Fisher Scientific, Waltham, MA, USA) or 10 pl Stealth RNAi Negative Control Duplexes as a control (Control; 10 μM, cat. no. 12935110, Thermo Fisher Scientific) using a narrow glass capillary tube (GC100 Tf-10, Harvard Apparatus, Holliston, MA, USA). For FRAP analysis, fully grown oocytes were co-injected with 10 pl siRNA of H3.1/3.2 (10μM) or Control (10 μM) and complementary RNA (cRNA) of eGFP-H2B (500 ng/μl). After injection, fully grown oocytes were washed with α-MEM (cat. no. 12571063, Thermo Fisher Scientific) without IBMX several times and incubated with α-MEM at 38°C for 18 h in an atmosphere of 5% CO_2_ and 95% air for *in vitro* maturation. After maturation, only MII-stage oocytes were selected and inseminated with spermatozoa obtained from the cauda epididymis of ICR male mice in human tubal fluid medium. After insemination, embryos were washed with KSOM medium several times, sperm, and cumulus cells were removed, and only fertilized oocytes were collected. The siRNA sequence against *H3*.*1/3*.*2* was 5′-GUGCGCGAGAUCGCGCAGGACUUCA-3′ (Supplementary Figure S1).

### Immunocytochemistry

The embryos were fixed with 3.7% paraformaldehyde (PFA) and 0.2% Triton X-100 in phosphate-buffered saline (PBS) for 20 min at room temperature, except for H3K4 immunostaining, in which embryos were fixed with 3.7% PFA in PBS for 20 min at room temperature and treated with 0.5% Triton X-100 in PBS for 15 min at room temperature. The embryos were washed with 1% bovine serum albumin (BSA) in PBS (PBS/BSA) and incubated with 1% BSA and 0.2% Tween 20 in PBS containing rabbit anti-H3K9me3 (1:500 dilution; cat. no. 07-442, Merck Millipore, Burlington, MA, USA), rabbit anti-H3K27me3 (1:100 dilution; cat. no. 07-449, Merck Millipore), mouse anti-γH2AX antibody (1:100 dilution; #07-164; Merck Millipore), rabbit anti-H2AK119ub antibody (1:2000 dilution; #8240; Cell Signaling Technology, Danvers, MA, USA), rabbit anti-H3K27ac (1:500 dilution; #C15410196; Diagenode, Belgium), rabbit anti-H3K4me3 antibody (1:500 dilution; 07–473, Merck Millipore), mouse anti-H3.1/H3.2 (1:500 dilution; CE-039B, Cosmo Bio, Tokyo, Japan) or rat anti-H3.3 (1:100 dilution; CE-040B, Cosmo Bio) antibodies overnight at 4°C. The embryos were washed with PBS/BSA and treated with secondary Alexa Fluor 488 anti-mouse (1:100 dilution; #A-11001, Thermo Fisher Scientific), Alexa Fluor 568 anti-rabbit (1:100 dilution; A10042, Thermo Fisher Scientific), Alexa Fluor 647 anti-rabbit antibody (1:100 dilution; A31573, Thermo Fisher Scientific), Alexa Fluor 647 anti-mouse (1:100 dilution; A21236, Life Technologies, Carlsbad, CA, USA) or Alexa Fluor 488 anti-rat (1:100 dilution; 4416S, Cell Signaling Technology, Danvers, MA, USA) antibodies for 1 h at room temperature. The embryos were washed with PBS/BSA and sealed in glass slides together with VECTASHIELD mounting medium with 4,’6-diamidino-2-phenylindole (DAPI) (H1200, Vector Laboratories, Newark, CA, USA).

In an experiment to quantify eGFP-H2B incorporated into the nuclei of two-cell embryos, the embryos were permeabilized with Triton X-100 before fixation with PFA as described in a previous report (28). The embryos were then washed with PBS/BSA and sealed in glass slides.

The samples were observed using an Olympus FV3000 laser scanning microscope (FV3000, Olympus, Tokyo, Japan). The fluorescence intensity levels of the H3.1/3.2, H3.3, H3K9me3, H3K27me3 and H3K4me3 were measured using Fuji software (29).

### BrdU assay

The timing of DNA replication was examined using a BrdU assay (28). Briefly, the embryos were incubated with KSOM containing 10 μM BrdU (cat. no. 90139520, Roche, Basel, Switzerland) for 1 h at 38°C. After incubation, the embryos were fixed with 3.7% PFA in PBS. The embryos were washed with PBS/BSA and incubated with 2N HCl containing 0.1% Triton X-100 for 1 h at 37°C. After treatment with HCl, the embryos were washed with PBS/BSA and treated with 0.1 M Tris–HCl (pH 8.5) containing 0.02% Triton X-100 in PBS for 15 min at room temperature for neutralization. The embryos were washed with PBS/BSA and incubated with mouse anti-BrdU antibody (1:100 dilution; cat. no. 11170376001, Roche) overnight at 4°C. Alexa Fluor 488 anti-mouse antibody (1:100 dilution; Thermo Fisher Scientific) was used as a secondary antibody. The embryos were washed with PBS/BSA, mounted on glass slides, and prepared for observation as described above.

### FRAP analysis

FRAP analysis was performed using an Olympus FV3000 laser scanning microscope as previously described (30). First, three pictures were taken at 5 s intervals using a 0.2% laser at an excitation wavelength of 488 nm, with a fluorescence intensity set to ∼2000, followed by photobleaching with a 3% laser at an excitation wavelength of 488 nm for 1 s. After photobleaching, 10 images were obtained at 5 s intervals with a 0.2% laser with an excitation wavelength of 488 nm. As an index of opened chromatin, the mobile fraction was calculated as described in a previous study (10).

### DNase sensitivity assay

A DNase sensitivity assay was performed as previously described (31), with slight modifications. Briefly, embryos were washed with 4 mg/mL polyvinylpyrrolidone (PVP; P5288, Sigma-Aldrich) in PBS (PBS/PVP) and then permeabilized with 0.2% Triton X-100 in PBS for 6 min at room temperature. The embryos were washed with PBS/PVP, transferred to DNase Reaction Buffer (RQ1 DNase 1× Reaction Buffer; M6101, Promega, Madison, WI, USA), and chromatin in the embryos was digested with 0.3 U/mL DNase in DNase Reaction Buffer (RQ1 RNase-Free DNase; M6101, Promega) for 15 min at 37°C. After digestion, the embryos were incubated with DNase stop solution (M6101, Promega) for 10 min at room temperature and fixed with 3.7% PFA in PBS for 15 min at room temperature. After fixation, embryos were labeled using TUNEL Enzyme (cat. no. 11767305001, Roche) and TUNEL label solution (cat. no. 11767291910, Roche), washed with PBS/PVP, mounted on glass slides, and prepared for observation as described above. The fluorescence intensity level was measured using Fuji software (29).

### EU assay to determine transcriptional activity

A 5-EU assay of the embryos was performed as described previously (32), with slight modifications. Briefly, the embryos were transferred to KSOM medium containing 2 mM 5-EU (C10330, Thermo Fisher Scientific) and cultured for 2 h. Then the embryos were fixed with 3.7% PFA for 20 min at room temperature. After fixation, the embryos were washed with PBS/BSA and treated with 0.5% Triton X-100 for 15 min at room temperature. Visualization of 5-EU incorporation with Alexa Fluor 594 was performed according to the protocol of the Click-iT RNA Alexa Fluor 594 Imaging Kit (C10330, Thermo Fisher Scientific). Embryos stained with Alexa Fluor 594 were mounted on VECTASHIELD mounting medium and samples were observed with a confocal microscope (FV3000). The fluorescence intensity level was measured using Fuji software (29).

### RNA sequencing (RNA-seq) analysis

MII-stage oocytes and embryos in each stage were sampled with ISOGEN and RNA extraction was performed as described previously (5,28). Reverse transcription and amplification of complementary DNA using the SMART-seq stranded kit (cat. no. 634444, Takara Bio, Shiga, Japan) according to the manufacturer's protocol. The amplified RNA-seq libraries were sequenced on a NovaSeq 6000 (150 nt paired-end sequencing).

### Hi-C analysis

Hi-C analysis was performed as previously described (33,34). Briefly, 20 embryos in each stage were treated with Acidic Tyrode's solution (MR-004-D, Merck Millipore) and Trypsin-EDTA (T4049, Merck Millipore) to remove polar body. The embryos were fixed with 2% formaldehyde (F8775, Sigma-Aldrich) for 15 min at room temperature, and then lysed with ice-cold cell lysis buffer (10 mM Tris–HCI, pH 8.0, 10 mM NaCl, 0.5% (v/v) NP-40; I8896, Sigma-Aldrich), 1% (v/v) Triton-X100 (cat. no. 0085111, Thermo Fisher Scientific), 1 Halt Protease Inhibitor Cocktail (cat. no. 78430, Thermo Fisher Scientific) for 15 min on ice. After lysis, the embryos were treated with 1 × NEB3 (B7003S, New England Biolabs, Ipswich, MA, USA) containing 0.6% sodium dodecyl sulfate (cat. no. 311-90271, Nippon Gene) for 2 h at 37°C. The embryos were treated with 5 U Dpnll (50000 U/mL; R0543M, New England Biolabs) at 37°C overnight and then treated with 5 U T4 DNA ligase (5 U/μl; EL0011, Thermo Fisher Scientific) for 4.5 h at 16°C. After ligation, the embryos were transferred to GenomiPhi v2 DNA sample buffer (cat. no. 25-6600-31, GE Healthcare, Chicago, IL, USA) and de-crosslinking was performed for 16 h at 65°C. Next, the genome was amplified using the Illustra GenomiPhi v2 DNA amplification kit (cat. no. 25-6600-31, GE Healthcare). The amplified genome was purified with SPRI beads (B23317, Beckman Coulter, Brea, CA, USA). The purified genome DNA was cleaved using a sonicator (M220, Covaris, Brighton, UK) to an average length of 500 bp, and the DNA library was prepared using the NEBNext Ultra II DNA Library Prep Kit (E7645S, New England Biolabs) for Illumina and sequenced using the Novaseq 6000 system for 150 nt paired-end sequencing.

### RNA-seq analysis

Adaptor and low-quality sequences were removed using fastp v 0.20.0 (35) with the options ‘-l 50 -w 24′. Ribosomal reads were removed using bowtie2 v2.2.4 (36). The remaining reads were mapped to the mouse genome (mm10) using STAR v2.6.0a (37), with the options ‘–outFilterMultimapNmax 1 –outFilterMismatchNmax 4′. Uniquely mapped read counts for each gene and repeat were calculated using featureCounts (subread v2.0.1) (38), and the transcripts per million (TPM) for each gene and counts per million (CPM) for each repeat were calculated using Python. Box plots, scatter plots, line plots, and a heatmap were drawn using R or Python. IGV v2.14.0 was used to map the locations of representative genomes.

To cluster the genes by expression patterns during the early and late two-cell stages, all genes were classified as follows. First, to exclude maternal transcripts that are expressed maternally and degraded after fertilization, we excluded genes whose expression continued to decrease during MII and the late two-cell stage. The remaining genes were defined as the group of genes transcribed from embryos. Next, genes whose expression increased during the early and late two-cell stages (log_2_[late two-cell/early two-cell] > 0.5) were defined as cluster I (increase); genes with little expression change (–0.5 < log_2_[late two-cell/early two-cell] < 0.5) were defined as cluster II (little change); and genes whose expression decreased during the early and late two-cell stages (log_2_[late two-cell/early two-cell] < –0.5) were defined as cluster III (decrease). Deseq2 v1.30.1 (39) was used to identify differentially expressed repeat elements between control and H3.1/3.2 KD embryos.

Previous data were used to analyze the localization of H3.1/3.2 and H3.3 (25). These data were processed using mouse genome (mm10) and Bowtie2 v2.4.2; the mapped reads were converted into bigwig files using the ‘bamCoverage’ function in deeptools v3.5.1 (40). The average read densities were plotted using bigwig files with the ‘computeMatrix’ and ‘plotProfile’ functions in deeptools v3.5.1.

The ratio of intergenic RNA reads to total RNA reads was calculated based on Ensembl GRCm38 (mm10) gene annotation. Intergenic regions including repeat elements were defined as regions at a distance >10 000 bp from genes, including non-coding genes and pseudogenes. Paired-end RNA reads were mapped to the reference sequences using STAR v2.7.9a (37) and their loci were compared to the reference gene annotation using bedtools v2.30.0 (41) and samtools v1.11/1.13 (42). Reads were considered intergenic if a template region of a read alignment was completely within the intergenic regions. Scripts for the analysis are available in a repository (https://zenodo.org/records/10465965).

### Hi-C data analysis

Hi-C reads were preprocessed using fastp v0.23.2 (35) and the resulting reads were mapped and summarized using distiller-nf v0.3.3 (https://doi.org/10.5281/zenodo.3350937). The Hi-C contact maps around TADs were aggregated, rescaled, and visualized using coolpup.py (43). Because the locations of TADs in two-cell stage embryos have not been reported, we used data obtained from mouse lymphoma cells (44), distributed as ‘GSE63525_CH12-LX_Arrowhead_domainlist.txt.gz’ in the Gene Expression Omnibus repository (accession no. GSE63525), which was previously used in an analysis of two-cell stage embryos (45). The locations of TADs are typically conserved among various cell types (44). TAD strength was defined according to the ‘get_domain_score’ function in coolpup.py, as previously described (33).

## Results

### Involvement of H3.1/3.2 in the formation of tight chromatin structure in two-cell embryos

**Figure 1.**
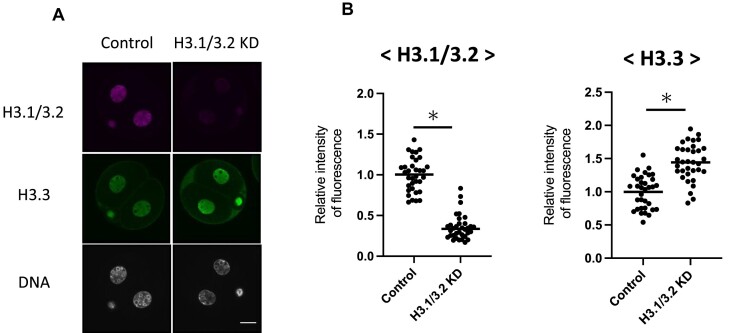
Knockdown (KD) of H3.1/3.2 by small interfering RNA (siRNA) in embryos. (**A**) Immunofluorescence images of H3.1/3.2 KD and control two-cell stage embryos stained with anti-H3.1/3.2 and H3.3 antibodies at 30 h post insemination (hpi). Scale bar = 20 μm. (**B**) Quantification of fluorescence signals in experiments in (A). Signal intensity levels of H3.1/3.2 and H3.3 were corrected with those of 4,’6-diamidino-2-phenylindole (DAPI). The value of the control embryos was set to 1 to calculate relative values. At least three independent experiments were performed for each experimental group, and more than 17 embryos in total were analyzed per group. Asterisks indicate significant differences (*P* < 0.05; Student's *t*-test).

The nuclear deposition of H3.1/3.2 increased between the early and late two-cell stages, during the transition from minor to major ZGA (24) (Supplementary Figure S2). Because the chromatin structure is closed during this period (10), the increase in H3.1/3.2 may be involved in this process in late two-cell stage embryos. To test this hypothesis, we knocked down H3.1/3.2 by microinjecting small interfering RNA (siRNA) against the common sequence of H3.1/3.2 (Supplementary Figure S1) into fully grown oocytes and allowed them to mature *in vitro*. The mRNA levels of H3.1/3.2, but not H3.3, significantly decreased in these oocytes in the metaphase II (MII) stage (Supplementary Figure S3A). After *in vitro* fertilization, H3.1/3.2 protein remained in both male and female pronuclei of H3.1/3.2 knockdown (KD) embryos, and H3.1/3.2 levels did not differ from those of control embryos (Supplementary Figure S3B). However, in the late two-cell stage, H3.1/3.2 levels were much lower in H3.1/3.2 KD embryos than in control embryos, whereas H3.3 levels were higher in H3.1/3.2 KD embryos (Figure 1). Thus, H3.1/3.2 levels were markedly decreased in the two-cell stage.

**Figure 2.**
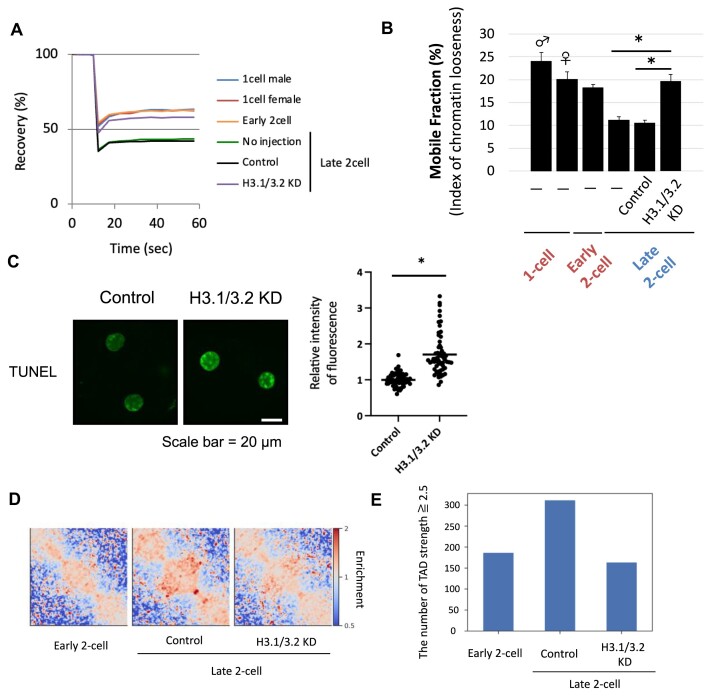
Chromatin incorporation of H3.1/3.2 plays an important role in the formation of open chromatin structure at the late two-cell stage. (**A, B**) cRNA encoding eGFP-H2B was co-injected with siRNA against H3.1/3.2 (H3.1/3.2 KD), control siRNA (Control) or none (–) into fully grown oocytes. Following maturation and fertilization *in vitro*, FRAP analyses were performed in the one-cell (10–12 hpi), and early (16–18 hpi) and late two-cell stages (30–32 hpi). Diagrams of the recovery curve and mobile fraction are shown on the left and right, respectively. ♂ and ♀ indicate male and female pronucleus, respectively. Three independent experiments were conducted to analyze 20 or more embryos in each group. Error bars indicate SE. Asterisks indicate significant differences (*P* < 0.05; Student's *t*-test). (**C**) Immunofluorescence images of H3.1/3.2 KD and control two-cell stage embryos stained with terminal deoxynucleotidyl transferase dUTP nick-end labeling (TUNEL) at 30 hpi to quantify fluorescence signals. The value of the control embryos was set to 1 to calculate relative values. Asterisks indicate significant differences (*P* < 0.05; Student's *t*-test). (**D**) Heatmap of topologically associating domain (TAD) strength in the Hi-C data for early and late two-cell control and H3.1/3.2 KD embryos. (**E**) Numbers of TADs with strength >2.5 in early and late two-cell stage (Control and H3.1/3.2 KD) embryos.

We performed fluorescence recovery after photobleaching (FRAP) analysis using eGFP-H2B to examine the effect of H3.1/3.2 KD on the establishment of a closed chromatin structure during the two-cell stage. First, we confirmed that the amount of incorporated eGFP-H2B was not affected by H3.1/3.2 KD (Supplementary Figure S4). Chromatin structure is extremely open in the one-cell stage, and closed during the two-cell stage (10), producing repressive chromatin (3,4). FRAP analysis showed that chromatin structure closed during the early and late two-cell stages and that chromatin remained open in H3.1/3.2 KD embryos in the late two-cell stage (Figure 2A, B). We also examined the effect of H3.1/3.2 KD on chromatin structure by conducting a DNase sensitivity assay in which DNA cleaved by DNase was detected with terminal deoxynucleotidyl transferase dUTP nick-end labeling (TUNEL) to determine chromatin accessibility. The TUNEL signal was higher in H3.1/3.2 KD embryos than in control embryos, suggesting that the formation of a closed chromatin structure was prevented by H3.1/3.2 KD (Figure 2C).

The topologically associating domain (TAD) is partially formed in the late two-cell stage (46). We analyzed TAD formation in H3.1/3.2 KD embryos via Hi-C analysis (33). Although the results showed that TADs increased between the early and late two-cell stages, this process was disrupted in H3.1/3.2 KD embryos in the late two-cell stage (Figure 2D, E, Supplementary Figure S5). We examined TAD strength and found fewer strong TADs (TAD strength > 2.5) in H3.1/3.2 KD embryos compared to control embryos (Figure 2E). An analysis of the inter- and intra-chromosomal contacts showed that >90% of contacts were intra-chromosomal in both control and H3.1/3.2 KD embryos.

Together, these results suggest that an increase in H3.1/3.2 is involved in the formation of closed chromatin structure and three-dimensional chromatin organization in the late two-cell stage, which suggests the establishment of repressive chromatin.

### H3.1/3.2 contributes to histone modifications in two-cell stage embryos

**Figure 3.**
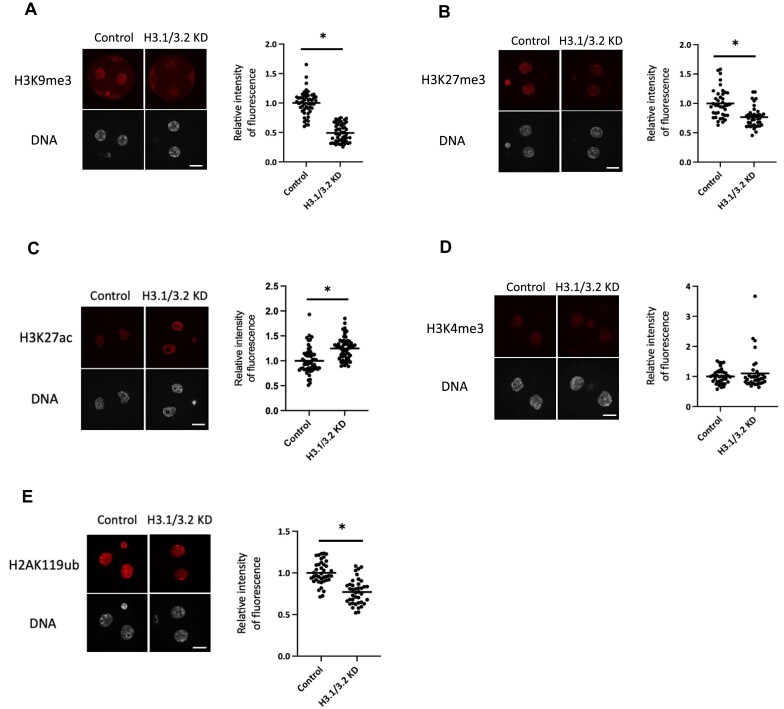
H3.1/3.2 are involved in histone modifications at the late two-cell stage. Left: immunofluorescence images of H3.1/3.2 KD and control two-cell stage embryos stained with the antibodies against (**A**) H3K9me3, (**B**) H3K27me3, (**C**) H3K4me3, (**D**) H3K27ac and (**E**) H2AK119ub at 28–30 hpi. Scale bar = 20 μm. Right: immunofluorescence quantification. Signal intensities of each histone modification were corrected against those of DAPI. The value of the control embryos was set to 1 and relative values were calculated. At least three independent experiments were performed for each experimental group, and more than 18 embryos were analyzed in total. Asterisks indicate significant differences (*P* < 0.05; Student's t-test).

H3 variants have unique modifications (18); among these, it appears that H3K9me2/3 and H3K27me3 play important roles in the formation of a closed chromatin structure, whereas H3K4me3 and H3K27ac are involved in an open structure. H3.1/3.2 are more likely to acquire H3K9me2/3 and H3K27me3, whereas H3.3 is preferentially modified by H3K4me3 and H3K27ac. Therefore, we examined these modifications in H3.1/3.2 KD embryos in the late two-cell stage. Immunocytochemistry analysis showed that the levels of H3K9me2/3 and H3K27me3 decreased in H3.1/3.2 KD embryos compared to control embryos (Figure 3A, B), which suggests that H3.1/3.2 contribute to higher levels of these modifications in the late two-cell stage. By contrast, H3K27ac levels increased in H3.1/3.2 KD embryos compared to the control (Figure 3C). H3K4me3 levels increased slightly but not significantly (Figure 3D). In addition, as it was reported that H2AK119ub is involved in repressive chromatin structure and gene repression at major ZGA (47,48), we examined H2AK119ub and found that its nuclear level was decreased by H3.1/3.2 KD (Figure 3E).

### H3.1/3.2 KD has no effect on the timing of DNA replication in the two-cell stage

H3.1/3.2 KD induced a change in chromatin structure in the late two-cell stage (Figure 2), and previous studies demonstrated that chromatin structure is involved in the timing of DNA replication (49,50). To investigate whether H3.1/3.2 KD affects the timing of DNA replication during the two-cell stage, we examined this process in H3.1/3.2 KD embryos by analyzing bromodeoxyuridine (BrdU) incorporation. However, no change was observed in the timing of DNA replication in response to H3.1/3.2 KD in the two-cell stage (Supplementary Figure S6).

### H3.1/3.2 are involved in gene expression pattern changes during minor and major ZGA

**Figure 4.**
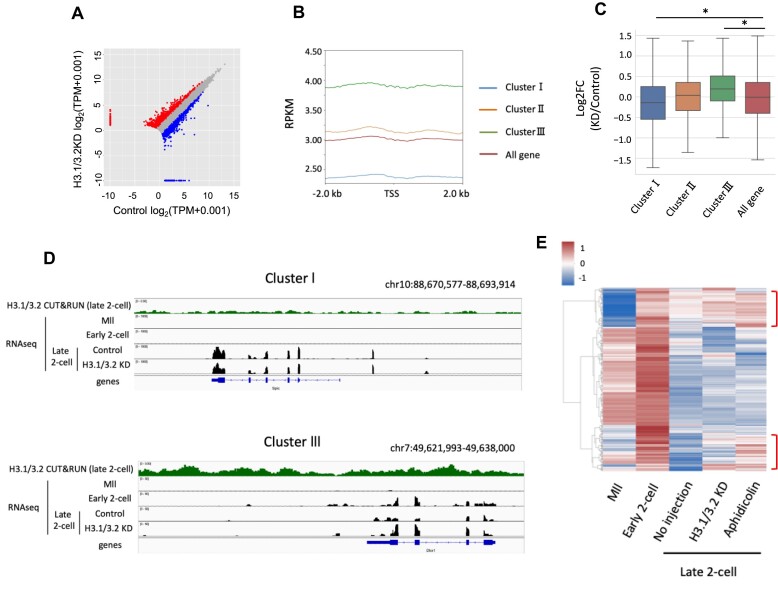
H3.1/3.2 regulate the change from minor to major ZGA**. (A**) Scatter plot of gene expression in H3.1/3.2KD and control embryos. Transcripts that increased or decreased by more than 2-fold in H3.1/3.2 KD embryos compared to control embryos are indicated in red or blue, respectively. (**B**) Amounts of H3.1/3.2 deposition in each cluster and whole genes in the late two-cell stage were plotted using deepTools. The Y axis indicates reads per million mapped reads (RPKM). (**C**) Changes in gene expression in the late two-cell stage by H3.1/3.2 KD in each cluster. Boxplots show the ratio of H3.1/3.2 to control. Asterisks indicate significant differences among genes (*P* < 0.05; Tukey–Kramer test). (**D**) Integrative Genomics Viewer (IGV) snapshot of the deposition of H3.1/3.2 and transcripts in a representative region around genes in clusters I and III. (**E**) Heatmap with hierarchical clustering of expression patterns of cluster III genes at Mll, the early and late two-cell (No injection, H3.1/3.2 KD, Aphidicolin) stages. Red brackets represent genes whose expression levels were increased by H3.1/3.2 KD and aphidicolin treatment.

Because H3.1/3.2 KD affected the formation of a closed chromatin structure in the late two-cell stage (Figure 2), we investigated their role in gene expression changes from minor to major ZGA. First, we performed a 5-ethynyl uridine (EU) label assay to determine whether H3.1/3.2 KD influences total transcription activity. There was no significant difference in activity between H3.1/3.2 KD and control embryos (Supplementary Figure S7). Next, we performed RNA-seq analysis in H3.1/3.2KD embryos; the results were reproducible according to the strong correlation between each replicate (Supplementary Figure S8). We found that H3.1/3.2 KD affected the expression of a number of genes in the late two-cell stage (Figure 4A). Next, we focused on the period from the early to late two-cell stage to investigate the involvement of H3.1/3.2 in the transition from minor to major ZGA by clustering genes based on changes in their gene expression patterns during this period (described in detail in Materials and Methods). Because maternal genes that were transcribed in oocytes persisted in two-cell stage embryos, we excluded genes that were transcribed in oocytes, but not in embryos, to strictly select genes transcribed in embryos. More than 90% of genes exhibiting significant expression changes due to H3.1/3.2 KD were genes transcribed in embryos (Supplementary Figure S9). We divided the resulting genes into three clusters based on their expression level changes from the early to late two-cell stages as follows: increased expression (cluster I), little change (cluster II), and decreased expression (cluster III) (Supplementary Figure S10). Then, we investigated the H3.1/3.2 deposition and expression levels of the genes in each cluster. We examined the deposition of H3.1/3.2 on genes in each cluster using previously published data on the genome-wide localization of H3.1/3.2 in late two-cell stage embryos (25). H3.1/3.2 were abundantly deposited on genes in cluster III that were repressed in the late two-cell stage; H3.1/3.2 were less abundant in cluster I genes, whose expression levels increase at that stage (Figure 4B, D). By contrast, opposite results were obtained for H3.3 (Supplementary Figure S11). Consistent with the clustering results, we detected a negative correlation (Spearman's *r* = −0.352) between H3.1/3.2 abundance at the late two-cell stage and expression changes from minor to major ZGA. After H3.1/3.2 KD, the expression levels of genes in cluster III were markedly increased in the late two-cell stage, whereas those in cluster I were not (Figure 4C, D). We also examined the effect of H3.1/3.2 KD on the expression of individual genes by real-time polymerase chain reaction (qPCR). *Eif1a*, *Zfp352* and *Zscan4d* are transcribed in the one- and early two-cell stages, and then decrease in the late two-cell stage (17,51). H3.1/3.2 KD reduced the degree to which the expression of these three genes decreased in the late two-cell stage (Supplementary Figure S12). Together, these results suggest that H3.1/3.2 cause the repression of a group of genes in the late two-cell stage.

### H3.1/3.2 are involved in DNA replication-dependent gene repression during the two-cell stage

Previous studies have reported that a group of genes whose expression is repressed at the late two-cell stage remained to be expressed after the inhibition of DNA replication by treatment with aphidicolin at the two-cell stage, suggesting that the second round of DNA replication is involved in the establishment of repressive chromatin (17,52,53). Because H3.1/3.2 are incorporated into chromatin in a manner dependent on DNA replication during the two-cell stage (24), H3.1/3.2 may be involved in DNA replication-dependent repression. Therefore, we analyzed cluster III genes that showed repressed expression in the late two-cell stage. We found that some of these cluster III genes were not repressed in H3.1/3.2 KD and aphidicolin-treated embryos at the late two-cell stage (Figure 4E). The expression levels of both sets of genes were clearly increased at the early two-cell stage after fertilization, demonstrating that they must be transcribed during minor ZGA. These results suggest that the nuclear incorporation of H3.1/3.2, which is dependent on the second round of DNA replication, is involved in the repression of a group of genes during the transition from minor to major ZGA.

### H3.1/3.2 are involved in the shift from promiscuous to regulated transcription during the early and late two-cell stages

**Figure 5.**
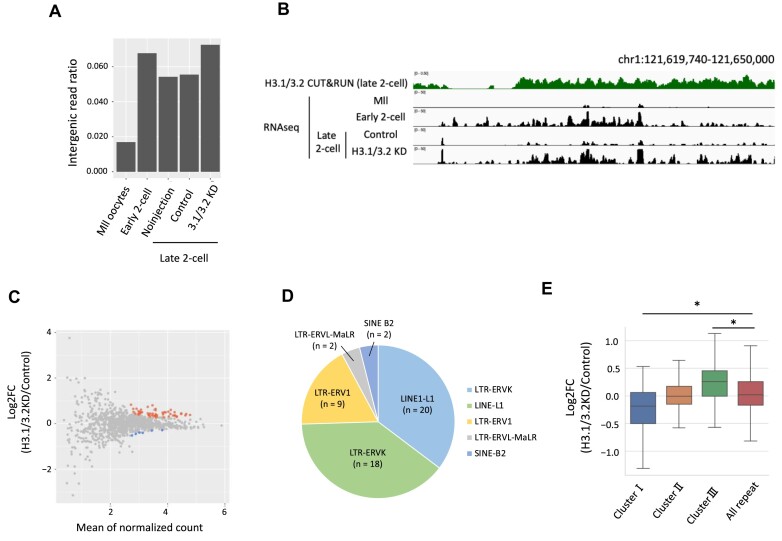
H3.1/3.2 repress transcription from intergenic regions in late two-cell stage embryos. (**A**) Expression ratios are from intergenic regions to the whole genome. (**B**) Snapshot of the expression of representative intergenic regions and localization of H3.1/3.2 at the MII and early and late two-cell stages. Snapshots cover intergenic regions. In the late two-cell stage, snapshots of embryos microinjected with H3.1/3.2 siRNA (H3.1/3.2KD) and control siRNA (Control) are shown. (**C**) MA plot of the expression levels of repeat elements in H3.1/3.2KD and control embryos. Transcripts that increased or decreased by more than 2-fold and adjusted *P* value was <0.05 in H3.1/3.2 KD embryos are indicated in red or blue, respectively. (**D**) Pie chart of the proportion of subfamily repeats with significantly upregulated expression in H3.1/3.2 KD embryos. (**E**) Changes in the expression of repeat elements by H3.1/3.2 KD in the late two-cell stage. Asterisks indicate significant differences among all repeats (*P* < 0.05; Tukey–Kramer test).

Because genome-wide transcription occurs during minor ZGA and declines during major ZGA (5), we investigated the involvement of H3.1/3.2 in transcription from intergenic regions. As reported previously, expression from the intergenic region increased in the early two-cell stage compared to the MII stage, and then decreased in the late two-cell stage (Figure 5A, B). However, in H3.1/3.2 KD embryos, transcription from the intergenic region was maintained in the late two-cell stage (Figure 5A, B), which suggests that H3.1/3.2 are involved in the transcriptional repression of intergenic regions from minor to major ZGA. Next, we analyzed the effect of H3.1/3.2 KD on the expression of repeat elements. In H3.1/3.2 KD embryos, 51 repeats were upregulated and 6 repeats were downregulated in late two-cell stage embryos (Figure 5C). Among the upregulated repeats, we detected the ERVK and LINE repeat subfamilies (Figure 5D). To examine the involvement of H3.1/3.2 in the expression of repeat elements, we classified the repeats into three clusters based on expression level changes between the early and late two-cell stages, as we had for genes (Supplementary Figure S13). Compared to controls, repeat expression was increased by H3.1/3.1 KD in cluster III but somewhat decreased in cluster I (Figure 5E). A previous study reported that LINE is transcribed in the one- and early two-cell stages and repressed in the late two-cell stage (5). H3.1/3.2 KD relieved the repression of LINE in the late two-cell stage (Supplementary Figure S12). These results suggest that H3.1/3.2 are involved in the repression of repeat elements in major ZGA.

### H3.1/3.2 KD has a detrimental effect on preimplantation development

**Figure 6.**
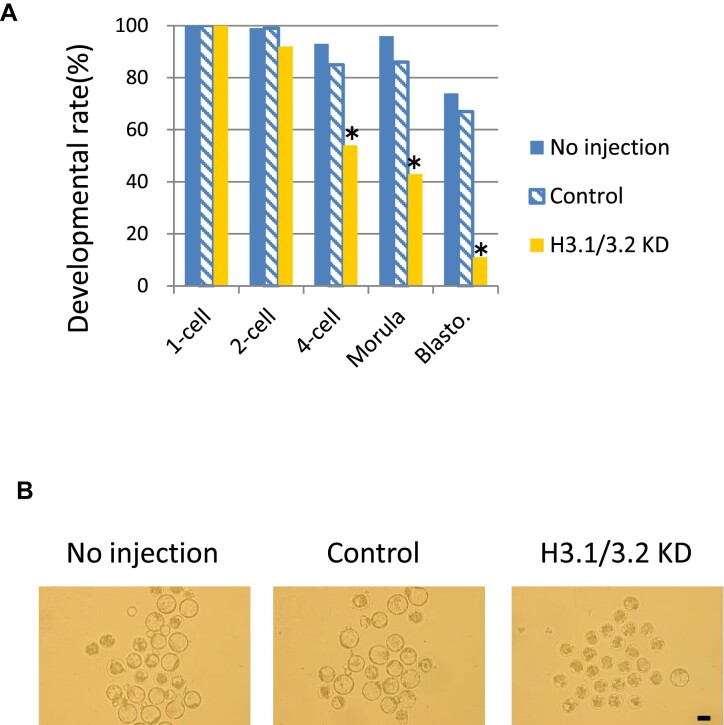
H3.1/3.2 are essential for preimplantation development. (**A**) Developmental rate of H3.1/3.2 KD embryos. Embryos injected with H3.1/3/2 siRNA, control siRNA (Control), or no injection were observed at 12, 24, 45, 72, and 96 hpi to evaluate development of the 1-, 2- and 4-cell, morula, and blastocyst stages. Asterisks indicate significant differences from both the no injection and control groups (*P* < 0.05; χ^2^ test or Fisher's exact test). Four independent experiments were conducted for each group and the data were accumulated. In total, 74 or more embryos were analyzed in each group. (**B**) Representative image at 96 hpi. Scale bar = 100 μm.

Finally, we investigated the significance of nuclear incorporation of H3.1/3.2 in preimplantation development. We observed the development of H3.1/3.2 KD embryos and found that H3.1/3.2 KD reduced the percentage of embryos that developed to the four-cell stage to 64% and to the blastocyst stage to 16% of the controls (Figure 6). This finding indicates that the incorporation of H3.1/3.2 during the two-cell stage is essential for preimplantation development.

## Discussion

After fertilization, the gene expression program is initiated during the mid-one-cell stage and first progresses during the two-cell stage, i.e. during the shift from minor to major ZGA (2,7,54). Although gene expression patterns and chromatin structure are dramatically altered during minor and major ZGA (5,55), the mechanism underlying these changes has not been elucidated. In this study, we demonstrated that increased H3.1/3.2 closed the chromatin structure and established regulated gene expression patterns in the late two-cell stage by repressing the expression of a set of genes and intergenic regions, including repeat elements that were promiscuously transcribed during minor ZGA.

Immunocytochemistry showed that the nuclear deposition of H3.1/3.2 occurred at low levels during minor ZGA and increased drastically at major ZGA (Supplementary Figure S2). By contrast, the nuclear deposition of H3.3 is constant during minor and major ZGA (Supplementary Figure S2). Our previous study showed that the expression levels of H3.1 and H3.2 were low at minor ZGA and increased at major ZGA, and that the incorporation efficiency of H3.1/3.2 was also lower compared with H3.3 at minor ZGA (24). These results suggest that both low incorporation efficiency at minor ZGA and increased expression at major ZGA are involved in the nuclear deposition dynamics of H3.1/3.2 during minor and major ZGA. Consistent with our previous report, a recent study using ribosome sequencing (56) showed that the transcription and translation levels of H3.1 and H3.2 increased more than two-fold between minor and major ZGA. Furthermore, the translation levels of histone chaperone CAF1 subunits (p150, p60, p48), which are involved in the incorporation of H3.1/3.2, also increased more than two-fold during minor and major ZGA (data not shown).

Developmental failure occurred in H3.1/3.2 KD embryos (Figure 6). This failure may be explained by abnormal gene expression in H3.1/3.2 KD embryos at the late two-cell stage, or by increased DNA damage. Previous studies have reported an association between chromatin structure and DNA damage (57–59), and we found that the foci of γH2AX, a DNA damage marker, increased in H3.1/3.2 KD embryos (Supplementary Figure S14).

Pioneering studies on ZGA regulation suggested that a change in chromatin structure is involved in the drastic change in gene expression from minor to major ZGA. Chromatin structure has been suggested to change from an open to closed state during the two-cell stage. Chromatin structure allows transcription without enhancers during the one- and early two-cell stages, when minor ZGA occurs, but then becomes repressive, requiring enhancers from the mid- to late two-cell stage, when major ZGA occurs. Under the repressive state, a functional enhancer is required for efficient transcription to relieve this repression (3,4,14,60). FRAP analysis revealed that the histone exchange rate became significantly slower between the early and late two-cell stage, suggesting that the chromatin structure became condensed during this period (Ooga *et al.*, 2016; Figure 2B). Our Hi-C analysis rarely detected TADs in the one- and early two-cell stages; however, TADs are detected in the late two-cell stage (46) (Figure 2D, E), which suggests that if chromatin structure is too immature to execute regulated transcription, promiscuous transcription can occur during the one- and early two-cell stages and is then established in the late two-cell stage. Therefore, in open (closed) chromatin structure at minor (major) ZGA, chromatin is decondensed (condensed), and transcriptionally permissive (repressive), and has undetectable (detectable) TADs. Our analyses showed that H3.1/3.2 KD caused the chromatin structure to remain open at the late two-cell stage (Figure 2) and decreased the levels of H3K27me3 and H3K9me3 (Figure 3A, B). H3.1 and H3.2 are abundant in transcriptionally inactive genome regions and enriched in H3K27me3 and H3K9me3, which are associated with the repression of gene expression and formation of heterochromatin (19,20). H3.1/3.2 KD also prevented the formation of TADs in the late two-cell stage (Figure 2D, E). Therefore, the incorporation of H3.1/3.2 would close the chromatin structure to establish repressive chromatin in the late two-cell stage. This hypothesis also explains the previous finding that DNA replication is associated with the change from enhancer-independent to enhancer-dependent gene expression regulation during the two-cell stage (15,52), because the incorporation of H3.1/3.2 into chromatin depends on DNA replication during the two-cell stage (24).

Under the influence of repressive chromatin, genes require specific transcription factors that result in regulated transcription during major ZGA. Several transcription factors involved in the activation of major ZGA genes were recently discovered, including DUX (61–64), NR5A2 (65), and the Obox family (66). RNA-seq data showed that H3.1/3.2 KD upregulated the expression of *Dux* and some *Obox* family genes (data not shown). Interestingly, *Eif1a*, *Zfp352*, and *Zscan4d* are known to be the target genes of DUX and OBOX (64,66). The upregulation of these target genes by H3.1/3.2 KD (Supplementary Figure S12) may be mediated by increased expression of *Dux* and *Obox*.

In the one-cell stage, a large proportion of genes are promiscuously transcribed at low levels. Although some of these genes are detrimental to early development, functional proteins are not generated due to inefficient splicing (5,67) and development is regulated at this stage by maternal mRNAs (68). However, these detrimental genes are expected to be repressed in the late two-cell stage, when splicing becomes functional and the maternal-to-zygotic transition of developmental regulation occurs. Our analysis suggests that cluster III genes contain some of these detrimental genes. H3.1/3.2 KD attenuated their repression (Figure 4C, D) and caused development failure (Figure 6). By contrast, cluster I genes, whose expression was upregulated in the late two-cell stage, were downregulated in H3.1/3.2 KD embryos in the late two-cell stage (Figure 4C). The rate of transcriptional activation during ZGA has been suggested to be limited by the amount of transcription factors (1). In H3.1/3.2 KD embryos, an open chromatin structure allows transcription factors to access many areas of the genome, including intergenic areas; thus, transcription factors have fewer opportunities to access cluster I genes, leading to the downregulation of cluster I genes in H3.1/3.2 KD embryos. Together, these results suggest that the switch from promiscuous to regulated transcription is important for proper gene expression, i.e. the expression of necessary genes and repression of unnecessary genes for early development, and that H3.1/3.2 regulate this change in transcriptional regulation.

The development in organism is thought to proceed following a gene expression program. In mice, minor ZGA is necessary for the occurrence of subsequent major ZGA (8). When transcription was inhibited transiently during the one-cell stage by 5,6-dichloro-1-β-d-ribofuranosyl-benzimidazole, a reversible transcription inhibitor, chromatin structure remained open, and promiscuous transcription still occurred when the embryos were free from the inhibitor in the late two-cell stage. These results suggest that the gene expression program proceeds in a stepwise manner during early preimplantation development. Our results demonstrate that H3.1/3.2 regulates this initial process of the gene expression program by establishing the closed chromatin structure to repress promiscuous transcription during the two-cell stage.

## Supplementary Material

gkae214_Supplemental_File

## Data Availability

All RNA-seq and Hi-C data used in this study have been deposited in the NCBI Sequence Read Archive (accession no. PRJNA1011510). Source codes are available at https://zenodo.org/doi/10.5281/zenodo.8113979.

## Supplementary data

Supplementary Data are available at NAR Online
