# Supplementary material for: H3.1/3.2 regulate the initial progression of the gene expression program
Source: Nucleic Acids Res. 2024 Apr 3;52(11):6158–70. doi: 10.1093/nar/gkae214 (PMC11194095; doi:10.1093/nar/gkae214)
Supplement: gkae214_Supplemental_File [file gkae214_supplemental_file.pdf]

Table S1. PCR primers

Name	Forward primer	Reverse primer	Tm (^{o}C)
H3.1	TGCAGGAGGCCTGTGA	TGGATGTCCTTGGGCATG	60
H3.2	TGCAGGAGGCGAGCGA	TGGATGTCCTTGGGCATG	60
H3.3a	CTCGGTGTCAGCCATCTTTCA	GAGCCATGGTAAGGACACCTC	60
H3.3b	GCTATTTGCTTGGTAGAAAAGGCT	TAAGCCCACCCATGAACCAC	60
Zcsan4d	CCTAGGGAGCTACCAGGGTT	AGGCTCCTGGCATGTTTGAA	60
Eif1a	AAGAAGTCTGAAGGCCTATG	CAGAGAACTTGGAAGGTAGC	60
Zfp352	AAAGCCTTGATCCTCAGGTG	GCCGAAGAGTTTTTCTGAGG	60
LINE-1	AGTGCAGAGTTCTATCAGACCTTC	AACCTACTTGGTCAGGATGGATG	60
Rabbit α-globin	GTGGGACAGGAGCTTGAAAT	GCAGCCACGGTGGCGAGTAT	60

Supplementary Fig. S1

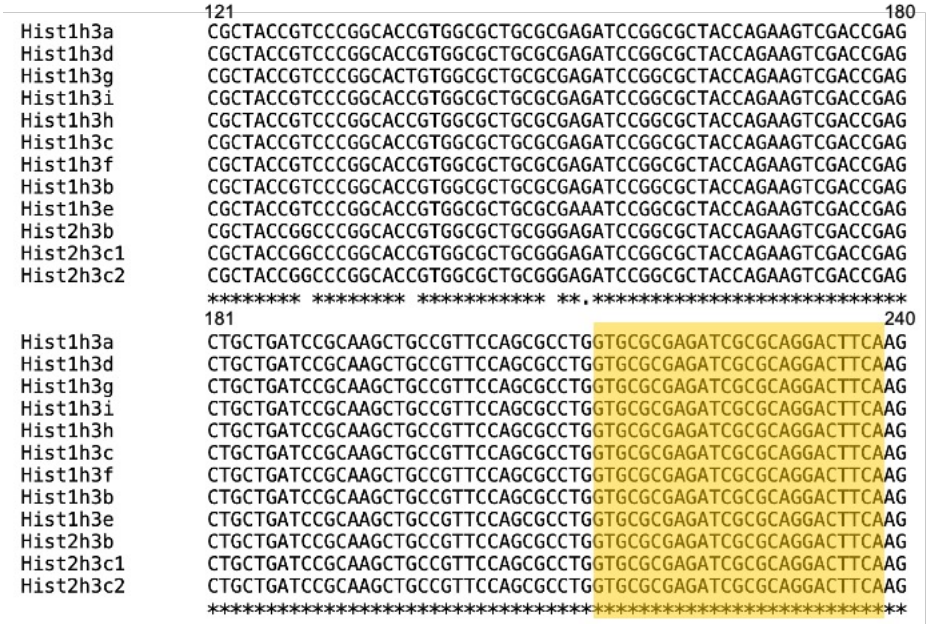

Supplementary Fig. S1. Target sequence of siRNA against the common sequence of H3.1/3.2. Parts of genes encoding H3.1 and H3.2 are shown. The siRNA target sequences are highlighted with yellow.

Supplementary Fig. S2

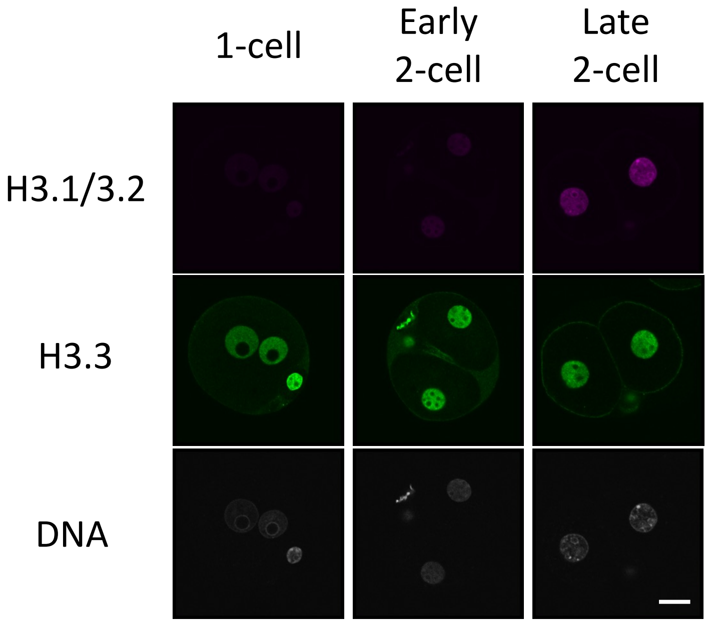

Supplementary Fig. S2. Nuclear deposition of H3 variants in one- and two-cell stage embryos. Embryos in the one- and early and late two-cell stages were collected at 11, 15, and 28 h post insemination, respectively, and then immunostained with anti-H3.1/3.2 and H3.3 antibodies. DNA was detected by DAPI staining. Two independent experiments were performed; representative images are shown. More than 10 embryos were analyzed in total. Scale bar = 20 μm.

Supplementary Fig. S3

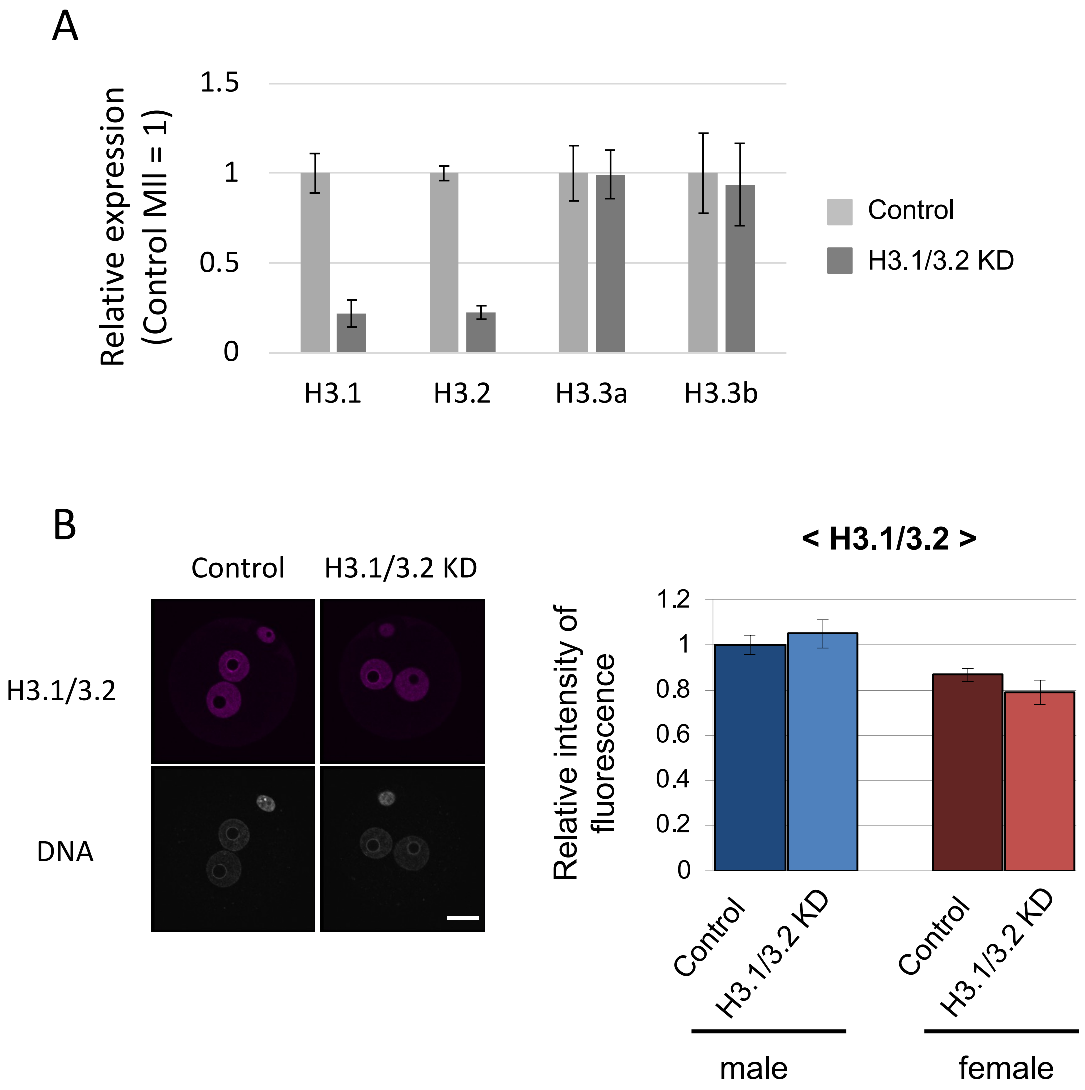

Supplementary Fig. S3. No effect of injection with H3.1/3.2 siRNAs on the levels of H3.1/3.2 proteins at the one-cell stage. (A) H3.1/3.2 KD and control MII stage oocytes were subjected to reverse-transcription polymerase chain reaction. Rabbit α-globin was used as an external standard. The value of the control embryos was set to 1 and relative values were calculated. (B) Left: H3.1/3.2 KD and control one-cell embryos (10 hpi) were subjected to immunostaining with anti-H3.1/3.2 antibody. Because the level of nuclear deposition of H3.1/3.2 is extremely low at the one-cell stage compared to the other stages, the laser power was enhanced to detect the immunofluorescence signal. Right: H3.1/3.2 signal intensity values were corrected with those of DAPI; those of male pronuclei in control embryos were set to 1 and relative values were calculated. At least four independent experiments were performed for each experimental group, and more than 25 embryos were analyzed in total. Error bars indicate SE.

Supplementary Fig. S4

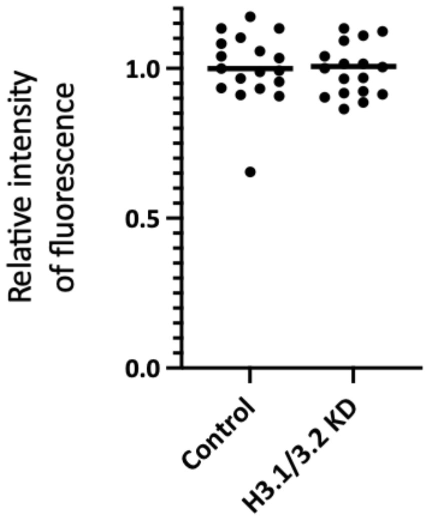

Supplementary Fig. S4. H3.1/3.2 KD has no effect on the incorporation of eGFP-H2B in two-cell stage embryos. Dot plot shows the quantified immunofluorescence of eGFP-H2B.

Supplementary Fig. S5

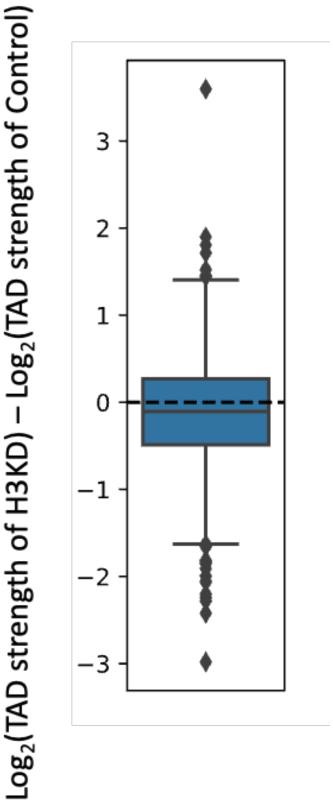

Supplementary Fig. S5. H3.1/3.2 influences the formation of TAD at the late two-cell stage. Box plot shows the difference in TAD strength between H3.1/3.2 and control.

Supplementary Fig. S6

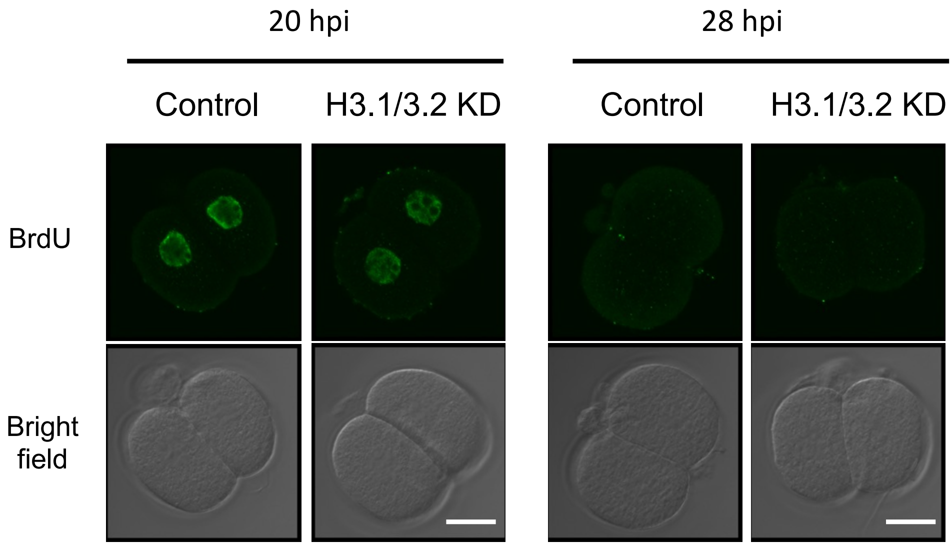

Supplementary Fig. S6. H3.1/3.2 KD has no effect on DNA replication in two-cell stage embryos. At each time point post-insemination (20, 28 hpi), the occurrence of DNA replication was examined by detecting bromodeoxyuridine incorporation. Two independent experiments were conducted to analyze a total of 17 or more embryos for each group and similar results were obtained in each experiment. Scale bar = 20 μm.

Supplementary Fig. S7

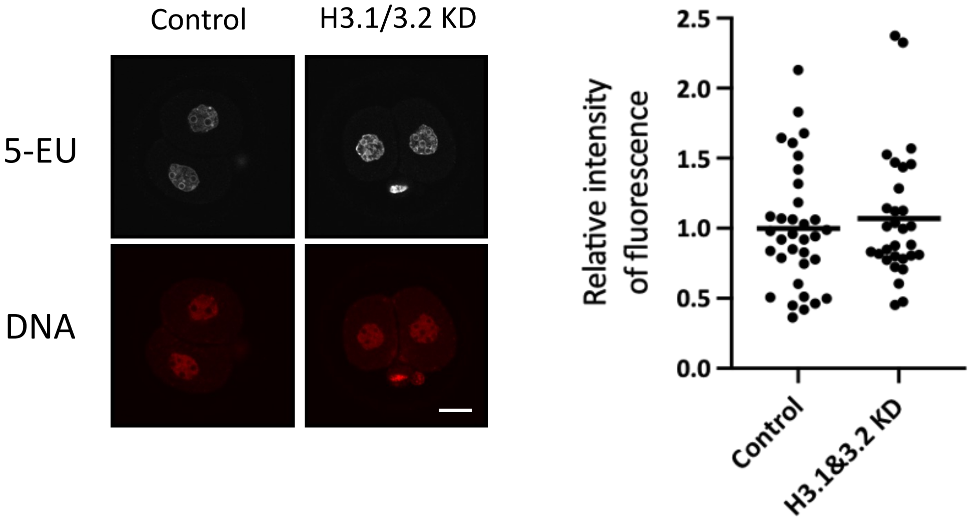

Supplementary Fig. S7. H3.1/3.2 KD has no effect on transcriptional activity in two-cell stage embryos. Left: Immunofluorescence images of ethynyl uridine (EU) in Control and H3.1/3.2 KD two-cell stage embryos at 30 hpi. Scale bar = 20 µm. Right: Quantification of immunofluorescence.

Supplementary Fig. S8

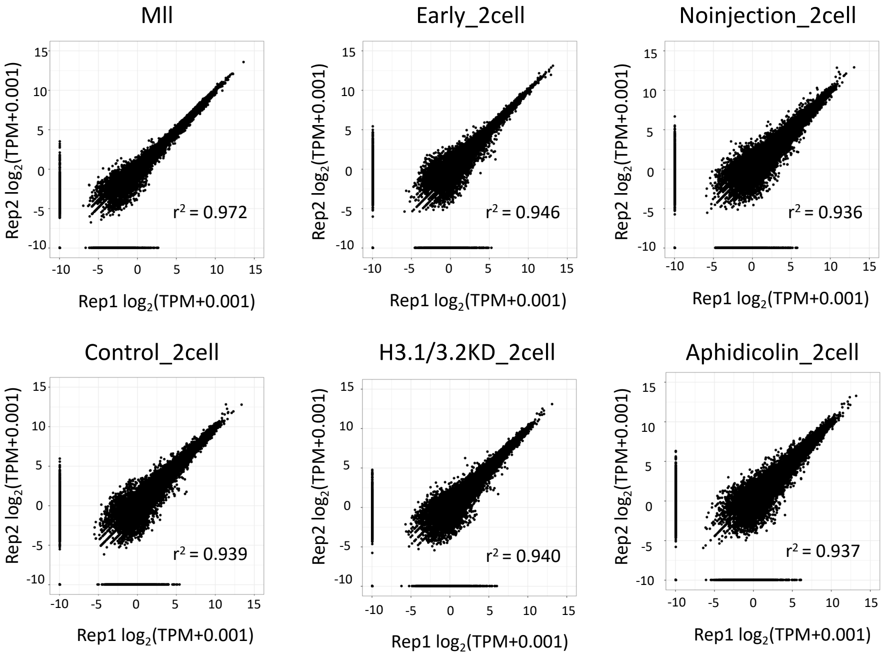

Supplementary Fig. S8. Validation of the RNA sequence data: comparison of sequencing replicates. Gene expression for each replicate. r^2 indicates Spearman's correlation coefficient.

Supplementary Fig. S9

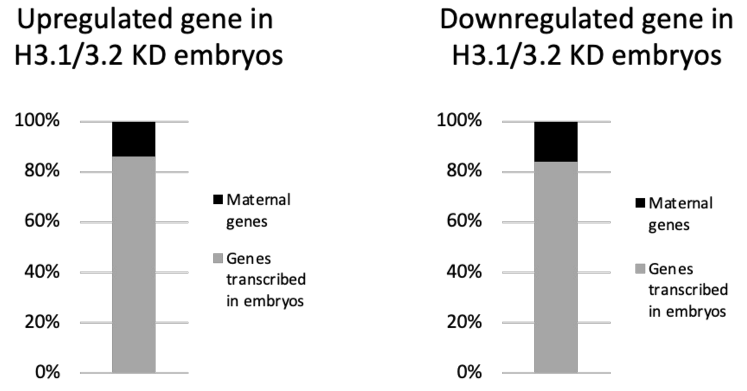

Supplementary Fig. S9. The ratio of maternal to zygotic genes up- and down-regulated by H3.1/3.2 KD. The genes which increased and decreased by 2-fold by H3.1/3.2 KD was defined as upregulated and downregulated, respectively.

Supplementary Fig. S10

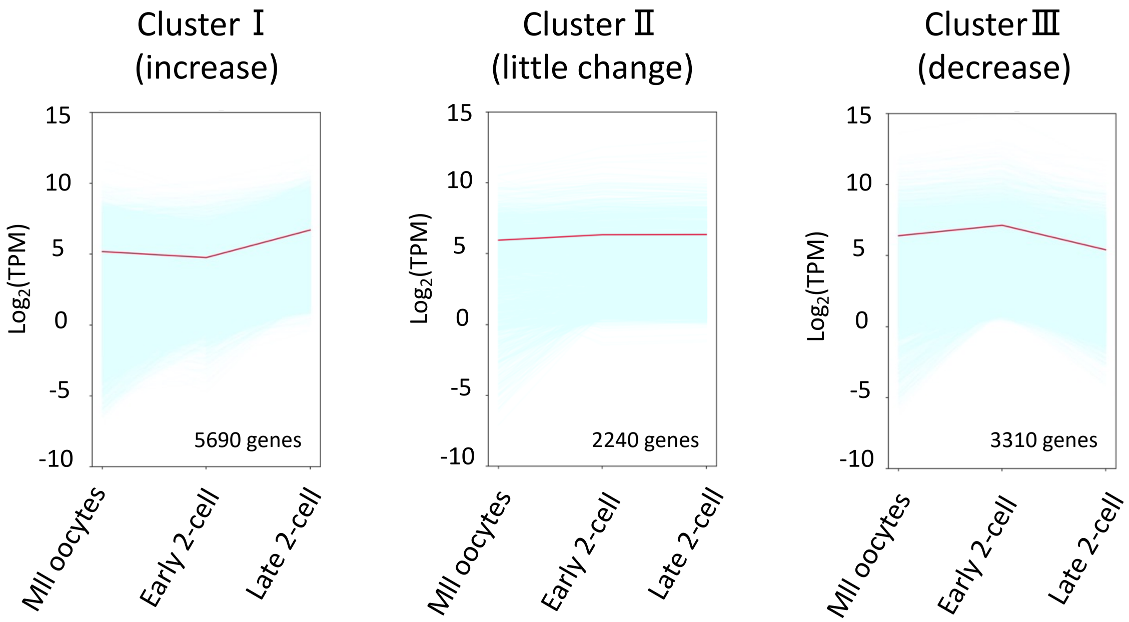

Supplementary Fig. S10. Clustering based on gene expression pattern changes during the two-cell stage. Genes are classified according to expression changes among MII stage oocytes and early and late two-cell stage embryos. The light blue line indicates the accumulation of expression change for individual genes; the red line indicates the average expression change for all genes.

Supplementary Fig. S11

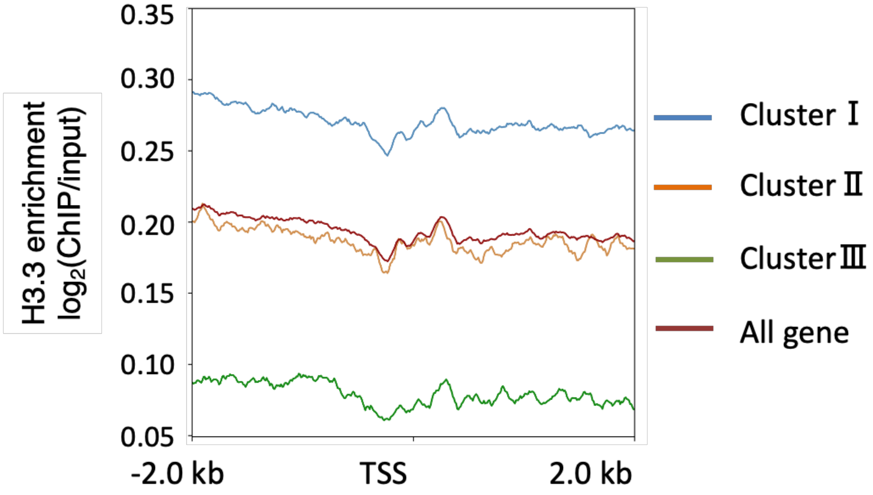

Supplementary Fig. S11. Amounts of H3.3 deposition in each cluster and whole genes in the late two-cell stage. H3.3 enrichment around TSSs of each cluster is plotted using deepTools.

Supplementary Fig. S12

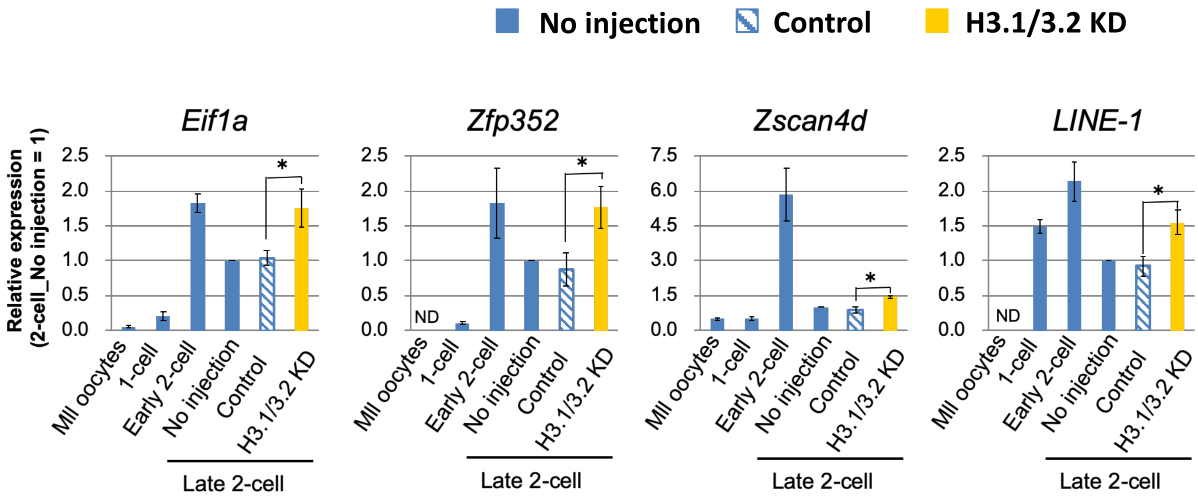

Supplementary Fig. S12. H3.1/3.2 regulate gene expression pattern changes during the early and late two-cell stages. Fully grown oocytes were injected with siRNA against H3.1/3.2 (H3.1/3.2 KD), control siRNA (Control), or no injection, and then matured and fertilized *in vitro*. Embryos in the one- and early and late two-cell stages were collected at 12, 18, and 32 hpi, respectively. Genes with expression levels that decreased between the early and late two-cell stage were examined in oocytes and embryos using RT-PCR. The value of two-cell stage embryos with no injection was set to 1 and relative values were calculated. Five independent experiments were conducted. Error bars indicate SE. Asterisks indicate significant differences ($P < 0.05$; Student's t-test).

Supplementary Fig. S13

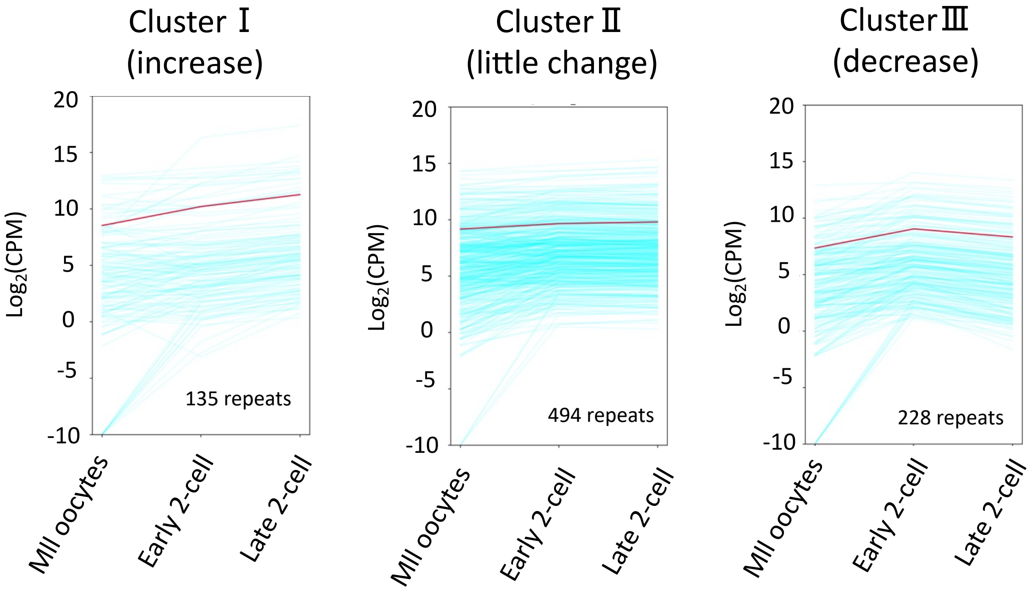

Supplementary Fig. S13. Clustering based on changes in repeat expression patterns during the two-cell stage. Repeat elements were classified according to expression changes among MII stage oocytes, and early and late two-cell stage embryos. The light blue line indicates the accumulation of expression changes for individual repeats; the red line indicates the average expression change for all repeats..

Supplementary Fig. S14

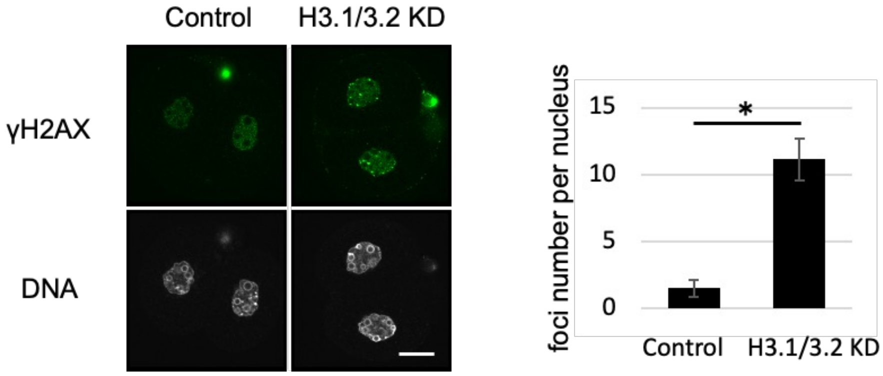

Supplementary Fig. S14. DNA damage in H3.1/3.2 KD embryos at the late two-cell stage. Left: Immunofluorescence images of γH2A.X in Control and H3.1/3.2 KD two-cell-stage embryos at 30 hpi. Scale bar = 20 µm. Right: Numbers of γH2AX foci in the nucleus in the immunofluorescence images shown in (A).
